# Evaluating a frontostriatal working-memory updating-training paradigm in Parkinson’s disease: the iPARK trial, a double-blinded randomized controlled trial

**DOI:** 10.1186/s12883-020-01893-z

**Published:** 2020-09-07

**Authors:** Magdalena Eriksson Domellöf, Lois Walton, Carl-Johan Boraxbekk, David Bäckström, Maria Josefsson, Lars Forsgren, Anna Stigsdotter Neely

**Affiliations:** 1 Department of Psychology, Umeå University, Umeå, Sweden; 2 Department of Social and Psychological Studies, Karlstad University, Karlstad, Sweden; 3 Umeå Center for Functional Brain Imaging (UFBI), Umeå University, Umeå, Sweden; 4 Department of Radiation Sciences, Umeå University, Umeå, Sweden; 5 Danish Research Centre for Magnetic Resonance, Centre for Functional and Diagnostic Imaging and Research, Copenhagen University Hospital, Hvidovre, Denmark; 6 Institute of Sports Medicine Copenhagen (ISMC), Copenhagen University Hospital Bispebjerg, Copenhagen, Denmark; 7 Department of Clinical Science, Neuroscience Umeå University, Umeå, Sweden; 8 Center for Demographic and Aging Research (CEDAR), Umeå University, Umeå, Sweden

**Keywords:** Working memory training, Updating training, Parkinson’s disease, Randomized controlled study, Cognitive training

## Abstract

**Background:**

Cognitive decline and dementia are common in Parkinson’s disease (PD). Cognitive deficits have been linked to the depletion of dopamine in the nigrostriatal pathway, but pharmacological treatments for PD have little evidence of improving or delaying cognitive decline. Therefore, exploring non-pharmacological treatment options is important. There have been some promising results of cognitive training interventions in PD, especially for improvements in working memory and executive functions. Yet, existing studies are often underpowered, lacking appropriate control condition, long term follow-up, a thorough description of the intervention and characteristics of the participants. Working memory updating training has previously shown to increase striatal activation in healthy young and old participants as well as dopaminergic neurotransmission in healthy young participants. In the light of dopamine dysfunction in PD, with negative effects on both motor and cognitive functions it is of interest to study if an impaired striatal system can be responsive to a non-invasive, non-pharmacological intervention.

**Methods and design:**

The iPARK trial is a double-blinded, randomized controlled trial with a parallel-group design that aims to recruit 80 patients with PD (during the period 02/2017–02/2023). Included patients need to have PD, Hoehn and Yahr staging I-III, be between 45 to 75 years of age and not have a diagnosis of dementia. All patients will undergo 30 sessions (6–8 weeks) of web-based cognitive training performed from home. The target intervention is a process-based training program targeting working memory updating. The placebo program is a low dose short-term memory program. A battery of neuropsychological tests and questionnaires will be performed before training, directly after training, and 16 weeks after training.

**Discussion:**

We expect that the iPARK trial will provide novel and clinically useful information on whether updating training is an effective cognitive training paradigm in PD. Further, it will hopefully contribute to a better understanding of cognitive function in PD and provide answers regarding cognitive plasticity as well as determining critical factors for a responsive striatal system.

**Trial registration:**

Clinicaltrials.gov registry number: NCT03680170, registry name: “Cognitive Training in Parkinson’s Disease: the iPARK study”, retrospectively registered on the 21st of September 2018. The inclusion of the first participant was the 1st of February 2017.

## Background

Parkinson’s disease (PD) is after Alzheimer’s disease (AD) the most common neurodegenerative disease with a prevalence of 1% in the population over 60 years of age [1], yet with some geographical variations [2]. The cardinal symptoms are motor impairments caused by depletion of dopamine in the brain, with severe depletion in the striatum [3]. In addition to the motor impairments, several non-motor functions are affected, of which cognitive deficits and dementia are among the most common. Some claim that up to 75% of the total PD population will eventually develop dementia [4] and effective treatments for cognitive impairment and dementia have a large clinical unmet need [5]. Prior to dementia, milder cognitive deficits are common, and already at the time of diagnosis, up to 42.5% of persons with PD are affected by Mild Cognitive Impairment (MCI) [4, 6, 7].

The cognitive deficits in persons with PD are heterogenic both in timing and in what cognitive functions are affected. According to the dual syndrome hypothesis proposed by Kehagia et al. (2013) there are two overlapping but separate cognitive systems affected in persons with PD. One syndrome with a possible cholinergic origin that presents with an early decline in visuospatial functions, semantic fluency, and episodic memory that is closely related to PD Dementia (PDD). The other is a frontostriatal syndrome affecting a larger part of the PD population with early deficits in executive functioning (EF) and working memory (WM) that is believed to be modulated by dopamine [8].

EF is a set of functions that control goal-directed behavior and include switching between sets, inhibiting and generating responses appropriately, and updating contents in working memory [9, 10]. WM updating is linked to the striatal dopaminergic pathway [11] and WM deficits have been linked to dysfunctions in these pathways in PD [12]. Brain-imaging studies have shown reduced transient activation patterns during WM updating in newly diagnosed persons with PD [13] and under-recruitment in an extensive brain network during updating in persons with PD-MCI [14]. WM updating training in healthy young and old individuals has previously shown that a period of updating training increased Blood Oxygen Level Dependent (BOLD) activity in the striatum, which correlated with training-related improved cognitive performance in both young and old [15]. Further, a corresponding effect of training on dopaminergic neurotransmission measured with Positron Emission Tomography (PET) using the radioligand raclopride was detected after the same training in young adults [16, 17]. This led to the question if the striatal system could also be responsive to this type of training in persons with documented decreases in dopamine availability, as in PD. A pilot study was conducted in one person with PD with the same working memory updating training paradigm as in the above-described study (Walton et al. submitted). Apart from significant improvements on the trained task, increased BOLD activity in the striatum was detected with fMRI after training, indicating the possibility of a responsive striatal system in early PD.

Although acetylcholinesterase inhibitors such as rivastigmine are indicated for PDD, there is no consistently successful pharmacological treatment for cognitive impairment in PD [18]. This patient group is already burdened by polypharmacy and therefore investigating non-pharmacological treatment options is crucial [19]. Recent systematic reviews on cognitive training in PD have shown evidence of improvements in overall cognition as well as in working memory, processing speed, and executive functions [20–24]. Most studies have had a broad cognitive approach [25–27] making it hard to know what aspects of training that is causing the improvement. Moreover, since most previous studies lack long-term follow-ups, it is impossible to know the long-term effects of training. Besides, some studies are underpowered and lacking appropriate control tasks, thorough descriptions of the intervention, and baseline characteristics of the participants. Baseline factors such as cognitive functioning, stage of PD, premorbid intelligence can have contributing effects on individual differences in training gain. Therefore, future studies need to include more participants, be theory-driven, and include more details of the cognitive profile, training intervention, and outcome measures [21].

Recently, different aspects of EF such as the shifting ability [28, 29] and the WM updating function [30] has been implemented in cognitive training regimes for PD with some promising results. Fellman et al. (2018) showed that persons with PD who participated in cognitive updating training had similar transfer patterns to that of healthy older adults (ie. improvement on WM tasks structurally similar to the trained tasks) and scored lower on self-assessed depression after training. This indicates the possibility of also improving psychological health with this kind of intervention.

Here we describe the iPARK study, a double-blinded randomized controlled trial investigating the effect of working memory updating training in persons with PD. The training paradigm used in iPARK has a strong neurobiological basis since it has previously been shown to increase striatal activation in healthy young and old adults as well as increased dopamine availability in young adults [15–17]. In the light of the dopamine dysfunction in PD, with negative effects on both motor and cognitive functions, it is of interest to study if an impaired striatal system can be responsive to a non-invasive, non-pharmacological intervention.

### Objectives

The iPARK trial aims to investigate the effect of a process-based cognitive training program that focuses on WM updating, compared to a low dose short-term memory paradigm, in persons with PD.

The specific research questions asked are:

1. Can working memory updating training lead to a more responsive frontostriatal system by improving the working memory updating ability in trained tasks?
2. Will there be improvements after training to untrained cognitive tasks (transfer effects)?
3. Will there be improvements in self-perceived everyday cognitive function and psychological health?
4. Will the observed improvements sustain 4 months after training?
5. Are there individual factors moderating the effects of training?

## Methods

The iPARK trial is registered at Clinicaltrials.gov with the study number: NCT03680170. The study was registered on the 21st of September 2018. Any changes to the protocol will be added to the trial registry at ClinicalTrials.gov. The study protocol complies with the Standard Protocol Items: Recommendation for Interventional Trials (SPIRIT) statement and with WHO:s Trial Registration data set.

### Study design and setting

**Fig. 1:**
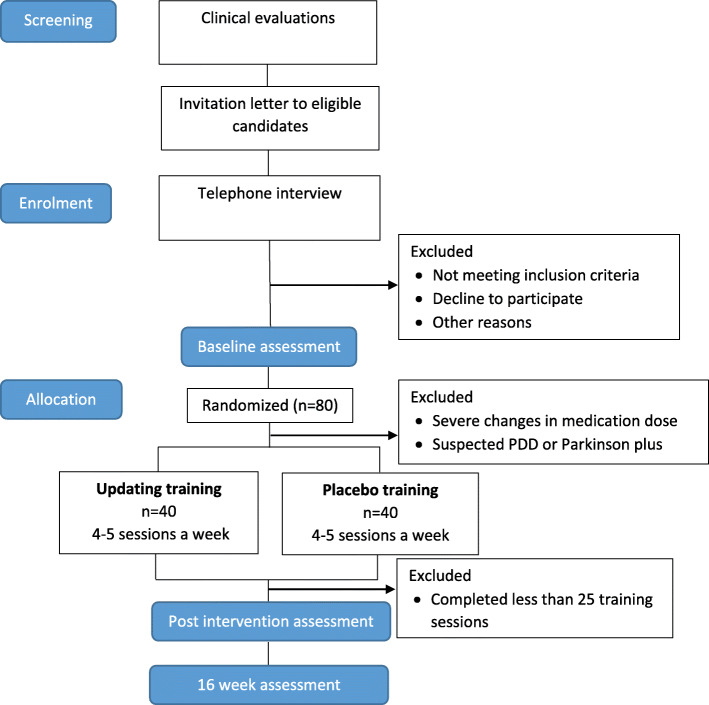
Flow chart

The iPARK trial is a double-blinded, randomized controlled trial with a parallel-group design. A minimum of 80 persons with PD will be included and randomly allocated to two intervention arms (see Fig. 1 for flow chart). The intervention of interest is a process-based training program focusing on WM updating. The placebo program is a low dose short-term memory program without the updating component. Both interventions are web-based performed at the participants’ home without supervision and will consist of 30 sessions over 6–8 weeks. The program includes one pre-training session and two post-training sessions, one directly after the training period and one after 4 months. The training program will be introduced by experienced research staff. Any obstacles will be monitored through telephone and/or e-mail.

### Participants

All participants will be recruited from the Department of Neurology at Umeå University Hospital, which represents the only Neurology clinic within a catchment area of around 142,000 inhabitants. A first screening will be based on clinical evaluations performed at the clinic during the previous year, performed by a study nurse. Only persons with a confirmed diagnosis of PD according to the United Kingdom Parkinson’s Disease Brain Bank (UKPDSBB) [31] with stable dopaminergic medication the past 3 months will be included in the study. Participation also requires being between stages one and three on the Hoehn and Yahr stage [32] and have a pathological Dopamine transporter (DAT) Single-photon emission computed tomography (SPECT) scan. Diagnoses of PD and Hoehn and Yahr assessments are established by neurologists specialized in movement disorders. Participants need a score of 24 or over on the Mini-Mental State Examination (MMSE) and are without dementia to be included in the study. All participants must have access to and be able to use a home-based computer with an internet connection. See Table 1 for inclusion and exclusion criteria. Participants with exclusion criteria such as dementia or advanced disease pre-defined through medical records will not be contacted for participation. Eligible participants will be contacted through an invitation letter where the basics of the study are explained. To check eligibility for participation the participants will be contacted by phone 1 to 2 weeks after the invitation letter by the test leader. During the initial phone call a short interview that covers computer literacy and accessibility as well as the time needed to complete the training will be included in the phone call. Individuals that agree to participate, fulfill inclusion criteria, and lack exclusion criteria will be invited to a pre-test and assigned a study number. If the presence of dementia or a disorder with atypical Parkinsonism is suspected after inclusion, the participant will be excluded. All other interventions and medication will be kept as stable as possible. Changes in treatment will be monitored throughout the study period.

**Table 1:** Inclusion and Exclusion criteria for the iPARK-trial

Inclusion criteria	Exclusion criteria
a) Diagnosis of Parkinson’s Disease according to United Kingdom Parkinson’s Disease Brain Bank (UKPDSBB) criteria b) Hoehn and Yahr stage I-III c) Pathological dat scan d) A score of 24 or over on the MMSE AND no Dementia d) Stable medication over the past 3 months e) Has access to and is able to use a homebased computer with internet connection.	a) Unstable medication b) Ongoing cognitive training c) Diagnosis of PDD d) Drug or alcohol abuse e) Other diseases of the central nervous system or other serious medical condition.

#### Randomization and blinding

Participants will be allocated to either the updating training or the placebo training after completing the baseline test. Computerized block randomization implemented by an independent statistician stratified by male/female and age (younger = < 65 and older = ≥66) will be performed. Participants in both groups will be given a code to get entry to the web-based training program in sealed envelopes at the end of the pre-test. The code will allocate the participant to either the updating training or the low dose short term working memory training. To avoid bias, participants will not be told which intervention they are undertaking and will be assigned to blinded assessors. Participants will also be instructed not to reveal any details about the training during contact with the assessors. To test the blinding, the assessor will report at the beginning of the first post-test which training program they believe the participant has been allocated to. At the end of the second post-test the participant will be asked which training program they think they have received. If the participant terminates his/her participation before the end of the study, the allocation will be unblinded.

#### Power and sample size estimates

The recruitment will continue until 40 individuals in each arm have gone through with the intervention and the first post-test. If participants drop-out during the study an additional participant will be recruited to maintain a sample size of 40 in each arm. Based on numbers of newly diagnosed cases in the region eligible for participation we expect the recruitment and testing will take three to four years.

The sample size was determined by a power calculation on the primary outcome measure (Letter Memory) based on data from older participants (mean(SD) age 69.3(4.9)) in one of our previous intervention studies [33]. Including 40 participants in each arm will generate a power of 0.9996, with a two-sided significance level of *p* = 0.05 based on the interaction effect of the previous study: (M*trained* post – *Mtrained* pre) - (*Mcontrol* post - *Mcontrol* pre) = 2.6, and a pooled standard deviation of 1.94.

### Intervention

The training program is web-based and conducted at the participant’s home four to five times a week for a total of 30 training sessions. The total training period will vary between 6 to 8 weeks. Each training session will be unsupervised and takes 20 min to complete. The training program consists of one criterion task and three training tasks that all aim to train the working memory updating function. The tasks will be performed in the same order for each training session. Participants will be asked how motivated they feel before each training session and how they perceived their ability to stay focused during training after each session. All items have a presentation time of 2000 ms with a stimulus interval of 1000 ms and the time to answer is unlimited. Feedback concerning numbers of correct answers are provided after each item as well as after each training task. The updating training program is adaptive to the participants’ performance and consists of three difficulty levels for all tasks except the criterion training task. When the participant performs at 80% or better at one level, they will advance to the next level for that specific task.

#### Criterion training task: letter memory running span [9]

**Fig. 2:**
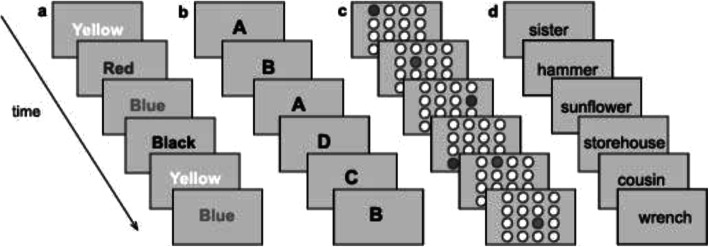
Illustration of the iPARK training program

In Letter memory (see Fig. 2b), 10 lists consisting of letters (A-D) presented serially with a random order in the center of the screen. The participants are asked to remember and report the four last letters after the presentation by clicking on four letters that are outlined at the bottom of the screen in the correct order. Each list consists of 5–14 presented letters. The list length is unknown for the participant. This task is also used to measure training gain throughout the training period to get a more detailed picture of learning curves during training. The number of correct recalled four-letter sequences (max = 10) and the number of correct individual letters (max = 40) will be used as outcome measures.

#### Training task 1: keep track task [9, 34]

In the “Keep track task” (see Fig. 2d) three trials consisting of 11–17 words presented serially in a random order in the center of the screen. Of the presented words; 9–15 belong to different semantic categories (animals, clothes, relatives, vehicles etc.), 2 words per list function as distractors and do not belong to any of the represented categories. Simultaneously semantic category boxes are displayed at the bottom of the computer screen. The participant is asked to mentally place the presented words in the box matching the semantic category of the word and report the last word presented from each category by typing in the word above each box after the presentation. Three difficulty levels are determined by varying the numbers of categories (boxes) presented, with low level = three boxes, medium level = four boxes; high level = five boxes and number of target words, low level = 9 targets; medium level = 12 targets; high level = 15 targets.

#### *Training task 2: Spatial running span* [9, 35]

In “the ball task”, (see Fig. 2c) the participants are presented with a 4 × 4 grid of circles. A circle is lit up in red in random, serial order and the participant is asked to remember and report the four last circles that are lit up by clicking on the circles in the correct order. Each list consists of 5–15 presented spatial locations. The three difficulty levels are determined by varying the list length, low level = 4–7 locations, medium level = 6–11 locations; high level = 5–15 locations.

#### *Training task 3: Color running span* [9, 35]

The final task is called the “the color task” (see Fig. 2a) five trials consisting of different colors (black, blue, yellow, and red) presented in a random, serial order for 2 s each in the center of the screen. The participants are asked to remember and report the four last colors after the presentation by clicking on four colors that are outlined at the bottom of the screen in the correct order. Each list consists of 5–15 presented colors. The three difficulty levels are determined by varying the list length, low level = 4–7 colors, medium level = 6–11 colors; high level = 5–15 colors.

#### Placebo program

The placebo program is a low dose short-term memory program without the updating component. Visually, it is identical to the updating-training except that only four letters/words/colors or spatial locations will be presented each round and will remain within the same difficulty level throughout the training period.

### Testing and outcome measures

All primary, secondary, and other pre-specified outcome measures will be assessed before training and twice after training (directly after training and after 16 weeks). The tests will be divided into primary outcome measures (criterion task), secondary outcome measures (near-, intermediate- and far-transfer tests), and other pre-specified outcome measures. The criterion test is measuring task-specific training gain with a similar task as in the training program. The near transfer tests are measuring the same abilit as trained (updating), but differ in terms of stimulus-respons mapping and task-format. The far transfer tests are measuring other untrained cognitive abilities. Adherence and compliance will be tested and monitored during training. For an overview of outcome measures and at which time point they will be tested see Table 2. All appropriate scales have been validated in Swedish.

**Table 2:** Outcome measures and demographics, including at which time point the data will be collected

	Outcome measures	Baseline	During intervention	Post-test	16 week follow-up
**Primary outcome measure**					
Criterion task	Letter memory running span	X	X	X	X
**Secondary outcome measures**					
Near transfer					
Updating	n-back (1,2 and 3 back)	X		X	X
	Digit memory running span	X		X	X
Intermediate transfer					
Perceptual and Psychomotor speed	Digit symbol	X		X	X
	Perdue Pegboard	X		X	X
Working memory	Digit span forward (WAIS IV)	X		X	X
	Digit span backward (WAIS IV)	X		X	X
	Digit span sequencing(WAIS IV)	X		X	X
	Spatial span	X		X	X
Inhibition	Stroop test (DKEFS)	X		X	X
Shifting	TMT A and B (DKEFS)	X		X	X
Far transfer					
Episodic memory	Buschke SRP	X		X	X
Fluid reasoning	Martices (WAIS IV)	X		X	X
**Other Pre-specified outcome measures**					
Subjective cognitive complaints	Prospective retrospective memory questionnaire	X		X	X
Depression and anxiety	Hospital Anxiety Depression scale (HAD)	X		X	X
Health status	Short form health survey (sf-36)	X		X	X
Sleep	Short version Karolinska Sleep questionaire	X		X	X
Function and well being	Parkinson’s Disease Questionnaire PDQ-39	X		X	X
Fatigue	Checklist Individual Strength questionnaire (CIS)	X		X	X
Impulsivity and Risk taking	Urgency, Premediation, Perseverance and Sensationseeking (UPPS)	X		X	X
	Balloon analog test	X		X	X
Adherence (task engagement)	Self-assessed motivation and ability to stay focused during training		X		
Compliance	Number of participants finishing within time frame		X		
Expectation	Expectation of improvement in certain tasks			X	X
Self-assessed improvement and adverse events	Evaluation of the training			X	X
**Demographics**					
Age	Age at baseline	X			
Gender	Gender	X			
Handedness	Lef/right	X			
Education level	Years of education	X			
Disease duration	Months	X			
Disease stage	Hoehn and Yahr stage	X			
Global cognition	Mini Mental State Examination (MMSE)	X			
Motor function	Unified Parkinson's Disease Rating Scale part III (UPDRS III)	X			
Disease laterality	Left/right	X			
Starting symptom and side	Symptom, Left/right	X			
Medication dose	LEDD	X		X	X
Vocabulary	Swedish four alternative multiple-choice synonym test (SRB 1)	X			
Cognitive status	MCI/NC	X			

#### Primary outcome measure (criterion task)

A letter memory running span task [9, 36] with a similar procedure as the task used during training will be conducted. As in the criterion training task, 10 lists of letters (A-D) are presented serially in the middle of the computer screen with a presentation time of 2000 ms and a stimulus interval of 1000 ms intervals with a small cross on the screen. The list lengths vary between 5 and 14 letters and are unknown to the participant who is asked to report the four last presented letters in the correct order after the presentation. In contrast to the training program the answers on the criterion task are timed, giving the participants 8000 ms to report an answer by using four adjacent keys on the computer keyboard with corresponding letters (A, B, C, and D) taped on the key. Between each list a circle is presented in the center of the screen for 6000 ms during which time the participant is asked to rest. Two parallel versions of the test will be used and counterbalanced between participants. The number of correct recalled four-letter sequences (max = 10) and the number of correct individual letters (max = 40) will be used as outcome measures.

#### Secondary outcome measures (near and far transfer)

The secondary outcome will be measured by a cognitive test battery consisting of a variety of tests.

##### Near transfer

Two near transfer tasks assessing updating consisting of n-back [37] and digit memory running span will be conducted. In n-back, digits are presented serially in the middle of a computer screen with a stimulus presentation time of 2 s with 1 s interstimulus intervals. The digits are presented in 27 sequences of 10 digits each divided into three different conditions of nine sequences: one back, two back, and three back. For each condition the assignment is to judge whether the number presented is the same as the number that appeared one, two, or three stimuli previously. The participants will be asked to report an answer for each presented number by using two adjacent keys on the computer keyboard with yes in green and no in red taped on to them. Hits minus false alarms (min 35-max 54) in each condition will be used to measure performance level and used as dependent measures.

In digit memory running span, a total of 10 lists consisting of digits presented serially with a random order in the center of the screen. The participants are asked to remember and report the four last digits presented by clicking on four digits that are outlined at the bottom of the screen in the correct order. Each list consists of 5–14 presented digits. The list length is unknown to the participant. All items have a presentation time of 2000 ms with a stimulus interval of 1000 ms, the time to answer is unlimited. The number of correct recalled four-digit sequences (max = 10) and the number of correct individual digits (max = 40) will be used as outcome measures.

##### Intermediate transfer

To measure perceptual and psychomotor speed, digit symbol from Wechsler Adult Intelligent Scale fourth addition WAIS-IV [38] and Perdue pegboard [39] will be used. In Digit symbol the participants are given a paper with a number coding key with numbers 1–9 associated with different geometric symbols on top of the assessment sheet. Further down on the assessment sheet numbers are shown together with empty boxes and the participants are instructed to draw as many geometric symbols in the empty boxes associated with the different numbers for 2 min. The total number of correct symbol drawings will be used as an outcome measure. Perdue pegboard is a test of fine manual dexterity of the upper limbs [40]. A white board with two cups containing small metal rods and two vertical rows of small holes is placed in front of the participant. The participant is asked to take a pin, one at a time from the right/left or both cup/s, and place in the holes starting at the top of the board on the right/left or both vertical line/s and repeat this during 30 s. Each condition is repeated 3 times. The outcome measure is the mean number of rods completed for each condition (right (or dominant)/left (or non-dominant)/both). The mean number of completed rods for the side most affected by PD will also be used as an outcome measure.

Working memory will be tested with digit span -forward, −backward, −sequencing, and spatial placing. Digit span forward, backward, and sequencing will be administered according to WAIS-IV [38]. Digits are presented orally with a rate of 1 sec per digit. The participants are instructed to remember the digits and report them back in the same order (forward), in reversed order (backward) and sequential order from one to nine (sequential). Two trials are presented for each span level. If the participant fails to report the digits in the correct order in both of the trials at one level the test is terminated. Both the total score and the highest span level (forward, backward, and sequential) will be used as outcome measures. Total min-max for each list (0–16) Total min-max for all lists (0–48), Span level min-max (forward 2–9, backward 2–8, sequential 2–9). Spatial working memory will be tested with an in-house developed computerized task named the “square” [41]. The participant will be presented with a 4 × 4 grid of squares. A square is lit up in red in random serial order and the participant will be asked to remember and report the squares that are lit up by clicking on the squares in the correct order after the presentation. Three trials are presented each span level starting with two spatial locations. If the participant fails to report the spatial location in the correct order in two of three trials at one level the test is terminated. Both the total score and the highest span level will be used as outcome measures. Total min-max (0–27), span level [2–10].

Inhibition will be measured with the Stroop task [42], administered according to standard procedures from the Delis-Kaplan Executive Function System (D-KEFS) [43]. The first test condition consists of 50 colored squares (red, green, and blue) printed on a sheet of paper that the participants are asked to name aloud. The second condition is to read 50 color words (red, green, and blue) written in black ink. In the third condition, there are 50 color words (“red”, “green” and “blue”), in red, blue, and green ink not matching the written word. The participant is asked to name the ink color. The instruction for all the lists is to complete each list as fast as possible without making any mistakes. Time in seconds to complete a list will be used as outcome measures. To get an inhibition score the third list minus the mean of list one and two will be calculated.

Shifting ability will be measured by the Trail Making Test (TMT) from D-KEFS [43]. The participants will be presented with a paper containing circles with letters and numbers in two different subtests. In the first test (TMT part 2) the participant will be asked to draw lines between circles in numeric order. In the second subtest (TMT part 4) the participants will be asked to draw lines between circles alternating between numbers and letters in numeric and alphabetic order (1-A-2-B-3-C…). Time to complete each subtest will be used as outcome measures. A flexibility score will also be used as an outcome measure calculated by subtracting TMT part 2 from TMT part 4.

##### Far transfer

Episodic memory and learning will be measured by an in-house developed program of free recall of 18 nouns administered according to Buschkes’ selective reminding procedure [44]. Initially participants will be presented with a list of 18 words presented serially on a computer screen with a stimuli presentation time of 5000 ms per item. The presentation is followed by a free recall test. Words not recalled successfully are presented to the participant verbally by the test leader with a 5 s stimuli interval on three subsequent trials. After each presentation the participant will be asked to recall the complete list again. A delayed recall performed 20–30 min after the learning phase will also be conducted. Three different lists of words will be counterbalanced among participants and test occasions. Total numbers of correct recalled words (max = 72) total numbers of correct recalled words for the trial with the best performance (max = 18) and total numbers of correct recalled words in the delayed recall (max = 18) will be used as outcome measures.

Fluid reasoning (nonverbal intelligence) will be measured by Matrices from WAIS-IV [38]. One (1 × 6) or two (2 × 2) dimensional matrices are presented to the participant. One section is missing in each matrix and the participant is asked to choose between one of five- alternatives that fit in the missing section. The session is terminated after three wrong answers in a row. There is a total of 26 items. Before the test two training items are presented where feedback is given to the participant. The total number of correct answers will be used as outcome measures (min-max: 0–26).

#### Other pre-specified outcome measures

##### Questionnaires

Subjective cognitive complaints will be assessed with the Prospective retrospective memory questionnaire (PRMQ) [45], a 16 item long questionnaire of memory slips in everyday life equally divided between questions about prospective memory problems such as “Do you decide to do something in a few minutes and then forget to do it” and questions about retrospective memory problems such as “Do you fail to recognize a place you have visited before”. Answers are given on 1–5 points Likert scale where 1 = never and 5 = very often.

The Hospital Anxiety Depression (HAD) scale is a 14 item long questionnaire equally targeting anxiety and depression, answers are given on a 0–3 Likert scale where 0 = never/no problems and 3 = always/a lot of problems. To measure general health status the short-form health survey (SF-36) will be used [46]. The questionnaire consists of 36 questions. The answers given varies between yes and no answers and Likert scales with three to six alternatives. The scale can both be used as a total score for general health and divided into physical function, role limitations physical and role limitations emotional, energy/fatigue, emotional wellbeing, social functioning, and pain. To assess sleep the short form of the Karolinska Sleep Questionnaire (KSQ-short) [47] will be given to the participant, a self-assessment form that measures different aspects of sleeping difficulties the past month. The KSQ-short form consist of six questions were the answers are given on a five-point Likert scale from 1 = never/very good to 5 = always/very bad. The Parkinson’s disease Questionnaire (PD-39) assessing function and wellbeing related to PD will be given to the participants. It consists of 39 questions were the answers are given on a Likert scale from 0 = never to four = always (cannot do it at all). Fatigue is assessed with the Checklist Individual Strength (CIS) [48], a self-assessment form consisting of 20 items rated on a seven-point Likert scale from 1 = yes, that is true to 7 = no, that is not true. Higher scores indicate more symptoms/problems for all measures.

##### Risk-taking

Impulsivity and risk-taking are assessed with Urgency, Premeditation, Perseverance, and Sensation Seeking (UPPS) and The Balloon Analogue Risk Task. UPPS is a self-administered questionnaire consisting of 45 questions rated on a 4 point Likert scale from 1 = completely true to 4 = completely false. The questions are divided into four factors of impulsivity related constructs [49]. Risk-taking is assessed with an in-house developed computerized test based on the Balloon Analogue Risk-taking Task (BART) [50]. The participant is presented with a balloon on the screen, which can be inflated by air by pressing a digital button with the left mouse key. The participant is told that the larger the balloon gets the more points are gained. The assignment is to get as many points as possible. If the balloon explodes, no points will be gained for that balloon. The computer program displays the size of the balloon visually and there are air puffing sounds for every time the participant inflates the balloon. There is also a pop-sound every time a balloon explodes. Each balloon is programmed to pop between 1 and 128 pumps. Information regarding the balloon breakpoint is not provided to the participant, who is told that the balloon can break at any time from the 1st pump. The participant can at any time stop pumping the balloon and collect their points by pressing the collect button. The outcome variable is average number of pumps for all the balloons that did not explode.

#### Compliance and adherence

Completing training on schedule, with 4–5 training sessions a week will be used as a measure of compliance to the study protocol. To minimize the dropout rate there will be supervision through telephone contact and e-mail/SMS regularly during the training period (see Table 3 for contact schedule). The online program will automatically record and store the time, date, Self-assessed motivation, and ability to stay focused during training, as well as the results for each training session.

**Table 3:** Adherence schedule with contact plan

Phone call	5–7 days
Mail or sms	2 weeks
Mail or sms	4 weeks
Mail or sms	6 weeks
Phone call	6–7 weeks

#### Dropouts and adverse events

Participants deciding not to follow through with the intervention are encouraged to participate in the follow-up assessments. All reports from the participants considered harmful or unwanted will be recorded, regardless of their relation to the training. Adverse events will be assessed, with telephone contact during the training period and with a questionnaire regarding self-assessed improvement and adverse events at the post-test and 16-week follow-up.

If participants report that the training makes them stressed to the extent that it affects their everyday life negatively or that it induces physical pain (headache, shoulder pain etc.). They will be advised by the test leader to terminate the intervention. Looking at previous cognitive training trials, serious adverse events are not expected to a great extent. This study is performed in close contact with the only neurology clinic in the area. Any unexpected findings will be discussed with the neurologist connected to the project (DB).

#### Potential moderating and mediating factors

Baseline factors such as age, gender, cognitive performance level, handedness, and education level will be collected at baseline. Information such as date of diagnosis, start of motor symptoms, Unified Parkinson’s Disease Rating Scale (UPDRS), Hoehn and Yahr stage, MMSE, disease laterality, starting symptom, laterality of symptoms at diagnosis and information regarding pathological DaTScan will be collected from the participants’ medical journal. Disease duration will be calculated as the time from date of diagnosis to pretest, while symptom duration will be calculated as the time from self-reported symptom onset to pretest.

#### Placebo effects due to expectations

The participants will be told that they are randomly allocated to either a memory-based intervention or an attention-based intervention. To reduce expectations in favor of one of the interventions both training programs are presented as equal alternatives. Both groups will get identical instructions on how the training program works and the research team does not speak in favorable terms for one intervention over the other. According to Boot et al. the effectiveness of the intervention could be questioned if the perceived intervention benefit correlates with improvement in that particular task [51]. To be able to measure expectations in relation to training gain, questions, whether the participant believes the intervention has led to an improvement in four different tests (Digit Symbol, Letter Memory, Matrices and n-back), will be assessed before each test during posttest (both directly after training and after 16 weeks).

### Data collection and management

Baseline clinical information (disease severity, global cognitive function, MCI, DAT-SPECT, brain structural MRI, Hoehn and Yahr stage, most affected side, etc.) are collected from the participant’s journals. All other data will be collected on paper forms and/or computer programs. All collected data will be entered into a joint database where each participant is given a study number. The personal identifier for each study number is stored in a locked compartment separated from the study-data. To ensure data quality an independent researcher will re-score 10% of the data.

After including 20 cases in each arm of the study. Interim analyses will be performed on the criteria test and the near transfer test pre and post as well as results during training to ensure data quality.

Results will be reported as scientific reports in peer-reviewed journals and international conferences. Attempts will be made to report back to the community through patient organizations.

#### Data analysis

A statistical plan will be developed before unblinding and data-analysis. The primary data analysis will be performed with an intention-to-treat principle.

Descriptive statistics will be presented by means and standard deviations for normally distributed data, while for variables with non-normal distributions medians and interquartile range will be presented. Frequencies and percentages will be presented for categorical data. Differences between the treatment groups at baseline will be tested using a multivariate analysis of variance.

The effects of the intervention will be assessed with the intention-to-treat principle where continuous outcomes will be analyzed using linear mixed models and categorical outcomes will be analyzed using generalized estimated equations. A random factor will be included for each subject. The fixed factors include treatment (updating or placebo intervention), test sessions (one, two, and three), and an interaction term between the treatment group and test session. If there are baseline factors that differ between the groups, they will be included as covariates with fixed effects in the models. The analysis will be conducted similarly regarding secondary outcomes. To assess if there are any moderating factors for training gains, sub-analyses will be performed with age, gender, disease severity, baseline cognitive function, and disease laterality added to the model in interaction with time. For randomly lost measures imputation based on predictive mean matching will be performed.

## Discussion

The iPARK trial is a randomized controlled double-blinded cohort study to assess the efficacy and long term effect of a computer-based training program performed from home in persons with PD. The intervention of interest is a process-based training focusing on working memory updating. The study will investigate if updating training will improve updating function in individuals with PD and if there are any transfer effects to other cognitive domains, self-perceived cognitive function, and quality of life. The study is designed to indicate any potential of a responsive striatal system also in persons with dopamine depletion. The overarching hypothesis is to provide support to that an impaired striatal system can be responsive to a non-invasive, non-pharmacological intervention of working memory updating which in turn can promote cognitive health and brain maintenance, which can contribute to reduced cognitive decline.

Cognitive decline is common in PD and is seriously affecting patients’ everyday life. Recent studies have also shown that PD complicated by cognitive dysfunction confers a shorter life expectance compared to PD with normal cognitive function [52]. The demand for non-pharmacological interventions is increasing and there is, although modest, growing evidence of the benefit of cognitive interventions in PD [20–22, 24]. While some studies have shown the efficacy of cognitive interventions in persons with PD, studies on newly diagnosed patients, excluding those with severe cognitive decline, are sparse.

The iPARK study aims to target patients with mild clinical symptoms of PD (no more than stage 3 on the Hoen and Yahr scale) without dementia. More advanced disease stages constitute other difficulties such as freezing of gate and levodopa-induced dyskinesia. Also, the dopamine depletion is more severe at later stages, which probably makes it harder to be stimulated by nonpharmacological interventions. Therefore, it is more likely that individuals early in the disease will have a more responsive striatal system. Also, a recent meta-analysis from the Cochrane Library found no evidence for any important cognitive improvement after 4 to 8 weeks of cognitive training in PD-MCI and PDD [53]. This indicates that cognitive interventions might be more suitable early in the disease when the cognitive decline is less pronounced.

This study has many strengths. Among them is the inclusion of an active control condition similar to the intervention of interest in all aspects except that the control condition does not include any updating in the training tasks. The majority of intervention studies use a no-contact or waiting list control and if a control-intervention is implemented it is often different from the training intervention, leading to a difference in expectations. To minimize the effect of expectation and to enable blinding, both study arms of the iPARK trial will be presented as equal interventions. Nevertheless, expectations of improvement will be measured before some post-tests to see if the two groups differ in that respect. Thereby, the iPARK trial will be able to answer if it is the updating itself that is causing the improvement. Furthermore, the iPARK trial will establish if there are any moderating factors for improved cognition after training in PD such as baseline cognitive function, age, gender, disease stage, and parkinsonian medication dose. Also, information on training results, motivation, perceived attention during training, time of day for each training session will be analyzed and provide more information as to why some participants improve, and others do not. Apart from measuring transfer to other cognitive tasks, the iPARK trial will also assess changes in psychological health and wellbeing. Finally, very few studies on cognitive training in persons with PD have performed any long term assessment, therefore the four-month assessment included in this study will provide knowledge on the long-term effect of training.

There are also potential limitations of the trial. The training will be unsupervised, which can make it difficult to maintain the motivation needed to complete the training. It can also be difficult to ensure participants are engaging in the training. The pros of an intervention being performed from home are that it can be implemented in everyday clinical practice with relative ease and low cost. To contribute to compliance with the intervention and the planned pre- and post-tests the participants will receive thorough information on what is expected of them during the study period. As a measure of engagement in the task, they will be given questions before and after training on motivation and execution in combination with logged results for each training session. Even though the similarity of the two intervention arms is a considerable strength, it also forms a potential limitation. The control condition could cause improvements in some of the tested functions, as well as psychological health and wellbeing, and thereby mask improvements of the updating training. Some participants might consider the control-condition too easy and drop-out while participants of the updating condition might consider it too hard. It is therefore important to register the reason why participants chose not to follow through with the intervention.

In conclusion, we expect that the iPARK trial will provide novel and clinically useful information about whether updating training is an effective cognitive training intervention for persons with PD. The iPARK trial will contribute to a better understanding of cognitive function in PD and evaluate a new non-pharmacological intervention for PD. If the training is successful, the next stage will be to conduct dopamine-PET and fMRI investigations before and after training as well as kinematic movement registration. Such further measurements will allow for a broader understanding of the effects of working memory updating. Thereby, providing knowledge about underlying mechanisms and answer if a period of updating training can stimulate increases of dopamine transmission also in PD.

## Trial status

The iPARK trial started in January 2017. Recruitment started in February 2017 and will continue until February 2023. The end date for the trial is July 2023. Any changes to the protocol will be made in the trial registry at ClinicalTrials.gov.

## Data Availability

The datasets generated and/or analyzed during the current study are not publicly available due to the ethical Review Act but are available from the corresponding author on reasonable request.

## Abbreviations

LEDD: Levodopa Equivalent Daily Dose
NC: Normal Cognition
PD: Parkinson’s Disease
AD: Alzheimer disease
MCI: Mild Cognitive Impairment
PDD: Parkinson’s Disease Dementia
EF: Executive functions
WM: Working Memory
BOLD: Blood Oxygen Level Dependent
PET: Positron Emission Tomography
UKPDSBB: The United Kingdom Parkinson’s Disease Brain Bank
MMSE: Mini-Mental State Examination
WAIS: Wechsler Adult Intelligent Scale
D-KEFS: Delis-Kaplan Executive Function System
TMT: Trail Making Test
PRMQ: Prospective retrospective memory questionnaire
HAD: Hospital Anxiety Depression
SF-36: Short form health survey
KSQ: Karolinska Sleep Questionnaire
UPPS: Urgency, Premeditation, Perseverance, and Sensation Seeking
BART: Balloon Analogue Risk-taking Task
PDQ-39: Parkinsonson’s Disease Questionaire
CIS: Checklist Individual Strength
UPDRS: Unified Parkinson’s Disease Rating Scale
